# Environmental legislation and capitalization of corporate environmental expenditure: Evidence from China

**DOI:** 10.1371/journal.pone.0277258

**Published:** 2022-11-03

**Authors:** Taiwei Wang, Yutao Chen

**Affiliations:** 1 School of Accounting, Southwestern University of Finance and Economics, Chengdu, Sichuan, P. R. China; 2 School of Accounting, Guizhou University of Finance and Economics, Guiyang, Guizhou, P. R. China; Universitas Nahdlatul Ulama Surabaya, INDONESIA

## Abstract

Although corporate environmental performance has received widespread attention and its economic consequences have been studied in the literature, little is known about the motivation of firms to choose different environmental strategies (corporate environmental expenditures). Constructing an indicator for the capitalization of corporate environmental expenditures, we explore corporate environmental strategies. This paper investigates the impact of environmental legislation on the capitalization of corporate environmental expenditures. By analyzing data on manual environmental expenditures of Chinese listed companies from 2011 to 2020, we find that implementing the new Environmental Protection Law (EPL) can enhance the capitalization of corporate environmental expenditures of high polluting firms. The effect is stronger for firms with higher external legitimacy pressures, such as those with greater public supervision and environmental enforcement. Firms with more internal resources are more likely to experience this effect, as evidenced by lower financing constraints and less managerial myopia. Further mechanistic studies suggest that implementing new EPL raises environmental media attention and perceived environmental risk among high polluting firms. This paper contributes to the environmental accounting literature by introducing a novel measure of corporate environmental strategy—the capitalization of corporate environmental expenditures. It investigates the effect of environmental regulation on the capitalization of corporate environmental expenditures to provide a reference for polluting firms’ corporate environmental strategy and the implementation of the EPL in China.

## 1. Introduction

Serious environmental pollution has become a significant problem for China as an emerging economy. The government has introduced a series of policies to push companies to control pollution to pursue "high-quality" growth. In particular, environmental legislation has become an important direction for China’s environmental governance reform. The new Environmental Protection Law (EPL), described as the "strictest ever" environmental law, was implemented in 2015 (MEE). It indicates the government’s desire to improve environmental quality by strengthening this environmental regulation on polluting firms through the rule of law. In response to the increasing concern about the implications of corporate environmental performance, many polluting firms consider embedding environment and sustainability into their financial decision-making [1], which is reflected in whether to expense or capitalize their corporate environmental expenditures.

Previous research in environmental accounting has concentrated on the relationship between corporate environmental performance and firm value [2–7]. Similarly, there is substantial empirical evidence that changes in the environmental performance of firms affect the behavior of a wide range of stakeholders, including shareholders, directors, creditors, analysts, and regulators [8–10]. However, there is relatively little empirical research on what motivates firms to choose different environmental strategies, such as corporate environmental expenditures. The purpose of this paper is to expand the literature on environmental accounting and legitimacy theory, and to examine how the capitalization of corporate environmental expenditures, as corporate environmental strategies, is affected by environmental legislation.

Distinct from previous studies, we use the ratio of environmental capital expenditures to the sum of corporate environmental expenditures to measure capitalization. Corporate environmental expenditures include both capital and expense components, as reported in the financial reports of Chinese listed companies. Combining two different corporate environmental expenditures to identify the capitalization of corporate environmental expenditures can distinguish this paper from prior similar studies. Prior research has primarily focused on environmental capital expenditures [4,5,11–13], which ignores environmental expense expenditures and fails to reflect the tradeoffs in firms’ environmental strategies comprehensively.

In terms of legitimacy theory, polluting firms need to improve their corporate environmental performance to adapt to the changing legislative environment [14] and satisfy stakeholders [15]. The cost of following mandatory environmental regulations for polluting firms is also high. Institutional theory suggests that institutional expectations do not apply uniformly to all firms, and firms can respond differently to these environmental regulation pressures [16]. Firms must take into account the potential benefits and costs when making environmental strategy decisions. Their environmental strategies are different: the firms focusing on preventing future pollution will more capitalize corporate environmental expenditures, while the firms focusing on the present end-of-pipe treatment of pollution will more expense corporate environmental expenditures.

Therefore, when faced with stronger regulatory pressure from environmental legislation, whether and how polluting firms adjust their capitalization of environmental expenditures as their environmental strategies, is the empirical question we address in this study.

We use a difference-in-differences research design to separate the effects of environmental legislation on the capitalization of corporate environmental expenditures from other confounding effects. To be specific, we compare corporate environmental expenditure capitalization for companies that are more affected by environmental legislation to corporate environmental expenditure capitalization for firms that are less affected by environmental legislation. The key to identifying high polluting firms is whether the firm is included in the National Specially Monitored Firms (NSMF) program implemented by the Ministry of Ecology and Environment (MEE). Those firms with high emissions would be included in the program of the year as high polluting firms.

To analyze the effect empirically, we hand collect corporate environmental expenditures data from the financial reports of Chinese listed firms. Specifically, we determine the extent of capitalization of corporate environmental expenditures by distinguishing between environmental capital expenditures (the amount of environmental "construction in progress") and environmental expense expenditures (the amount of "pollution fees"). Our primary sample consists of 6,295 firm-year observations listed on the Chinese stock market during 2011–2020. We find that environmental legislation is significantly associated with the increased capitalization of corporate environmental expenditures. For example, treatment firms increase their capitalization of corporate environmental expenditures by 7.4% compared to control firms. These results are robust to a series of robustness tests. Our results suggest that environmental legislation enhances the management of corporate legitimacy and thus increases the capitalization of corporate environmental expenditures, which is consistent with the existing literature [13].

In addition, we further investigate the impact of external legitimacy pressures and potential internal resources for firms, and we conduct four cross-sectional analyses to identify conditions under which environmental legislation may differentially affect the capitalization of corporate environmental expenditures. First, we find that for firms in regions with stricter local environmental enforcement, environmental legislation helps increase the capitalization of corporate environmental expenditures. Second, for firms with higher public supervision, environmental legislation has an incremental effect on the capitalization of corporate environmental expenditures. Third, the positive relationship between environmental legislation and the capitalization of corporate environmental expenditures is more significant for firms with lower financing constraints. Finally, for the effect of the manager, there is evidence that managers’ characteristics influence corporate decision-making [17]. We find that capitalization increases more significantly for firms with lower managerial myopia. In summary, the evidence from cross-sectional tests suggests that the effect of environmental legislation on the capitalization of corporate environmental expenditures appears to be more vital for firms with higher external legitimacy pressures and more internal resources. These findings further support our contention that environmental legislation raises the importance of legitimacy management and that firms with more resources become more sustainable and greener.

We further investigate the mechanisms by which environmental legislation increases the capitalization of firms’ environmental expenditures. Prior research has argued that stakeholders, such as the media, are essential third parties for monitoring firms’ environmental performance and influencing legitimacy [18,19]. Therefore, we use the number of environmental-related news to measure environmental media attention and find that high polluting firms experience increased media attention following environmental legislation. Second, we examine whether firms in the treatment group are more likely to perceive their environmental risks. We find that these firms quickly recognize increased environmental risks by increasing the environmental-related descriptions in the "Management’s Discussion and Analysis" section of their annual reports. Empirical evidence suggests that environmental legislation can strengthen firms’ understanding of legitimacy, encouraging them to capitalize corporate environmental expenditures and choose more proactive corporate environmental strategies.

This paper contributes to that literature in several ways. First, how firms engage in sustainable development has attracted public attention. However, little is known about what factors motivate firms to pursue different environmental strategies. Changes in corporate environmental performance may affect the behavior of a wide variety of stakeholders, including creditors, shareholders [8], analysts, managers, and local communities [20]. Our study provides evidence that environmental legislation promotes firms’ adoption of proactive environmental strategies. In addition, we add to the environmental accounting literature by identifying an important, but previously little-identified, measure of corporate environmental strategy—capitalization of corporate environmental expenditures. The study of this issue is also of considerable importance to regulators and other stakeholders concerned with corporate environmental performance. This is evidenced by the Chinese government’s continued introduction of environmental regulations to enhance the requirements for corporate legitimacy (MEE). Given the worldwide quest for comprehensive Sustainable Development Goals (SDGs, United Nations) and the various benefits of environmental measures by polluting firms, we believe it is timely and important to examine the factors influencing firms’ environmental strategy choices.

Second, legitimacy theory suggests that firms need to adapt to the changing legislative environment in order to increase their organizational legitimacy [14]. Firms are rewarded for increasing legitimacy, and social stability by conforming to institutional pressures [16,21,22]. Thus, as a proxy for institutional pressure, the relationship between environmental regulation and corporate environmental performance has received considerable attention from researchers. In spite of this, empirical evidence from the literature on this relationship is mixed [13,23]. A plausible explanation for this contradictory result is that firms can comply with environmental regulations by strategically selecting different environmental expenditures. Existing studies usually focus on the impact of environmental regulations on environmental capital expenditures. After considering environmental expense expenditures, the potential impact of capitalizing environmental expenditures has not been explored. We extend the study of the impact of environmental regulation on firms’ environmental performance to a new measure of environmental strategy, capitalization of corporate environmental expenditures.

Last, our paper also has some practical implications. Our findings will help firms understand the effects of environmental legislation more fully. By focusing on the potential benefits and costs of environmental regulations on polluting firms, we examine how environmental legislation affects the capitalization of corporate environmental expenditures. This firm-level environmental strategy decision directly reflects the net effect of such regulations. By providing evidence on the factors that influence the environmental strategy decisions of polluting firms in China, this paper’s findings may assist in promoting more proactive environmental actions by polluting firms.

The rest of this paper is organized as follows. Section 2.1 looks at the institutional background, Section 2.2 reviews the related literature, and Section 2.3 develops the hypothesis. The following describes the sample selection, database, variable measurement, and regression models in Section 3. Section 4 reports and discusses our main tests and additional tests. Finally, Section 5 concludes the remarks.

## 2. Institutional background, literature review, and hypothesis development

### 2.1 Institutional background

China has experienced impressive economic growth since economic reforms were initiated in the late 1970s. China’s rapid economic growth has not come without a price. When economic development was prioritized, it inevitably resulted in uneven growth in China, leading to significant social and environmental costs. A World Bank report published in 2016, The Cost of Air Pollution: Strengthening the Economic Case For Action, shows that the welfare loss in China due to air pollution amounts to 10.9% of GDP. The Chinese government is gradually recognizing the serious environmental problems, acknowledging the severe deterioration of China’s environment, and calling for a system of environmental management and legislation (MEE). The old Environmental Protection Law of the People’s Republic of China (EPL), which was enacted in 1979, is the primary legal basis for environmental management in China. The old EPL suffers from inefficient implementation [24]. As a response to environmental challenges and international governance regimes, introducing a new EPL is imperative.

In 2015, the new EPL, known as the "strictest ever", was officially implemented in China. For the first time, environmental protection was written into the law as a basic state policy. The new EPL highlights the government’s regulatory function and the public’s right to monitor it. The government hopes to improve environmental quality by strengthening environmental regulation through the rule of law. The new EPL includes three significant aspects: strengthening environmental supervision, increasing pollution penalties, and public participation.

The implementation of the new EPL is an exogenous shock to polluting firms. Firms face a series of enhanced environmental regulatory measures, including administrative regulation (e.g., increased enforcement powers of environmental protection departments), financial penalties (e.g., increased fine caps), public disclosure (e.g., mandatory disclosure of environmental information), and public scrutiny (e.g., environmental NGOs can sue polluting firms directly). Compared to low polluting firms, high polluting firms are more strongly affected by the new EPL. Because of their high emissions and "original sin", regulators and the public will be more concerned about whether their emissions are illegal. Accordingly, high polluting firms will be more affected by the new EPL.

### 2.2 Literature review

In this study, we examine the relationship between environmental legislation and the capitalization of corporate environmental expenditures. Research examining the relationship between environmental regulation and firms’ environmental performance has been developed for decades. As the institutional basis of environmental regulation, environmental legislation is critical to the impact of firms’ environmental performance.

A series of studies have shown contradictions in the effects of environmental regulation on firms’ environmental performance. Prior literature suggests that environmental regulation, especially the improvement of environmental laws, has a positive impact on firms’ reduction in pollution emissions [25–27] and increases in environmental investments [13,28]. For example, Adams and Kuasirikun [28] state that environmental laws and regulations in many nations cause firms to increase capital spending and operating costs for environmental protection initiatives. Zhang et al. [13] find that environmental regulation helps firms to boost environmental capital expenditures. In general, environmental regulations can motivate firms to adopt more proactive environmental strategies and make more investment in environmental expenditures to reduce future environmental risks. As a result, corporations may provide investors with information on environmental capital expenditures as a sign of their proactive environmental strategy [4]. Furthermore, firms set themselves apart from their competitors by demonstrating their improved environmental performance to investors [29].

Nevertheless, some studies have argued that environmental regulations do not necessarily improve firms’ environmental performance. As a consequence of environmental regulation’s ineffectiveness in reducing wastewater emissions, it had no significant impact on the productivity of surviving firms [23]. Wu et al. [30] found that the locational choice of newly polluting firms in water pollution management planning was a shift from coastal provinces with stringent environmental requirements to western provinces with lenient environmental requirements. As a result of environmental regulations, multinational firms are more likely to transfer pollution than their efforts to improve the environment because of short-term profit considerations [31]. A reflection of the extant literature suggests that the impact of environmental regulation on firms’ environmental performance is inconsistent.

Existing studies have used a variety of methods to measure the environmental performance of firms. There are indicators of individual emissions, like individual greenhouse gas indicators, which support a partial description of environmental performance [32]. In addition, there are other methods to determine the environmental performance management of firms. For example, some studies have used questionnaire mediation methods to assess firms’ environmental performance management systems by interviewing their executives and employees [33,34] and have explored the factors influencing firms’ environmental strategies. Some studies calculate green input-output efficiency [35]. From the perspective of environmental accounting, prior research has focused solely on environmental capital expenditures [4,5,11–13], ignoring environmental expense expenditures and failing to adequately reflect tradeoffs within firms’ environmental strategies.

In summary, the existing literature examines environmental regulation and corporate environmental performance in different contexts. Some studies have analyzed the relationship between environmental regulation and firms’ pollution levels. Some studies have investigated whether environmental regulation is an influencing factor for firms’ environmental expenditures. It should be noted that heterogeneity in the literature regarding firms’ environmental expenditures has been neglected. Firms’ environmental capital expenditures are different from their environmental expense expenditures, reflecting different environmental strategies. Therefore, we discuss the relationship between environmental legislation and the capitalization of corporate environmental expenditures from an analytical perspective and test its significance. We further identify the explanatory factors behind this potential relationship.

### 2.3 Hypothesis development

We argue that a firm’s environmental performance can be evaluated in terms of inputs and outputs. A firm’s environmental expenditures measure environmental performance on the input side [12], reflecting a firm’s environmental strategy. Unlike the existing literature that focuses only on environmental capital expenditures, we reflect the corresponding environmental strategy by distinguishing between firms’ environmental capital expenditures and environmental expense expenditures. In terms of environmental accounting, environmental capital expenditures refer to expenditures that qualify for asset recognition, such as green improvement technology renovation, desulfurization projects, and denitrification projects. They provide meaningful information for assessing future environmental performance [11].The firm’s environmental capital expenditures are investments that will prevent future environmental risks and indicate a proactive environmental strategy. Conversely, expense environmental expenditures are recognized as corporate expenses, which are mandatory costs in order to compensate for past ecological damage. For example, corporate sewage charges represent a negative corporate environmental strategy, meeting only the minimum regulatory requirements. This paper argues that the higher the capitalization of firm’s environmental expenditures, i.e., the greater the ratio of environmental capital expenditures to total corporate environmental expenditures, the more proactive the firm’s environmental strategy.

Following our previous discussion of the relationship between environmental regulation and firms’ environmental performance, we take an improvement strategy view of the impact of environmental legislation on firms’ environmental performance, by assuming that environmental legislation increases the capitalization of firms’ environmental expenditures. First, firms’ tradeoffs between the potential benefits and costs of environmental strategies are ultimately reflected in their capitalization of environmental expenditures. According to legitimacy theory, environmental legislation increases the level of environmental regulation faced by polluting firms, and firms are required to adapt to an increasingly demanding legislative environment [14]. However, the cost of polluting firms adhering to mandated environmental regulations is equally substantial. As compared to environmental expense expenditures, the direct cost of adopting environmental capital expenditures is higher. According to institutional theory, institutional expectations do not apply uniformly to all firms. A firm’s response to environmental regulation demands can differ depending on its institutional expectations [16]. After the new EPL implementation, firms must consider the potential advantages and costs of adopting different types of corporate environmental expenditures.

Second, high polluting firms may perceive higher environmental risks after implementing the new EPL. The new EPL implementation has increased the penalties for environmental violations, and high polluting firms should expect future environmental risks to rise. They will increase their environmental capital expenditures for risk prevention. Environmental legislation motivates firms to consider environmental impacts in their strategic decisions. Indeed, previous research has found that polluting firms incorporate the goals and measures of stakeholders, including the government, into the design of their strategic performance management systems [33]. To decrease future environmental risks, firms are more inclined to go green and sustainable by increasing their capital environmental investments, increasing the share of environmental capital expenditures in corporate environmental expenditures, and adopting more aggressive environmental strategies.

Finally, the new EPL implementation has increased corporate environmental information disclosure, facilitating media access to environmental information about high polluting firms and improving media oversight. The new EPL requires that all polluting firms regularly disclose their emissions information and accept media and public oversight. Media coverage of firms’ environmental behavior can trigger public concern, which plays a critical role in public oversight of environmental governance. The studies documented that negative media coverage of firms, especially environmental coverage, leads to a decline in firm value, negative investor reactions [19,36], and reputation decline [18]. As a result, after the new EPL implementation, the environmental information of high polluting firms will be more likely to be noticed by the media than that of low polluting firms. Firms will avoid media coverage of their environmental damage behavior, which will be detrimental to their reputation creation and market value. Thus, firms will increase the capitalization of corporate environmental expenditures and adopt more proactive environmental strategies, making it difficult for the media to criticize their environmental performance.

Following these arguments, we state our first hypothesis as follows.

***H1*: *High polluting firms will increase the capitalization of corporate environmental protection expenditures*, *subsequent to the new Environmental Protection Law (EPL) implementation*.**

With the implementation of the new EPL, the change in capitalization of corporate environmental expenditures may be more significant for firms in areas with high legitimacy pressure. To further explore the effect of legitimacy pressure on the above relationship, we investigate the effect of environmental enforcement and public concern on the capitalization of corporate environmental expenditures of high polluting firms.

After the new EPL implementation, its effects depend on the enforcement of local governments. Compared to the old EPL, the new EPL focuses on increasing the penalties for environmental violations. It increases the amount of penalties and punishes environmental violations continuously. Additionally, it strengthens administrative penalties for enterprises and their principals, including production suspension and seizure.The governance effect of the new EPL depends not only on its severity, but also on the actual enforcement by local governments [30]. The enforcement of environmental regulations by local governments is insufficient [24], and local governments have varying levels of enforcement. Therefore, the regulatory effect of environmental legislation can only be significant in areas with strict environmental enforcement.

As mentioned above, after implementing the new EPL, high polluting firms in areas with more vigorous local environmental enforcement will be subject to stricter environmental regulations. High polluting firms will face more frequent pollution monitoring, higher fines for environmental violations, and a higher risk of administrative penalties for managers, subsequent to the new EPL implementation. Strict environmental regulations will induce high polluting firms to reduce pollution emissions and increase environmental capital expenditures [13] to reduce future environmental risks. Therefore, we propose the following hypothesis:

***H2a*: *The association between high polluting firms and the capitalization of corporate environmental expenditures is more significant for firms in areas with stricter local environmental enforcement than for firms with weaker local environmental enforcement*, *subsequent to the new Environmental Protection Law (EPL) implementation*.**

The new EPL reduces the cost for the public, especially environmental NGOs, to monitor polluting firms and improves public participation in environmental monitoring. As mentioned earlier, the new EPL promotes the firms’ disclosure of environmental information, which in turn reduces public resistance to obtaining accurate environmental information. In addition, the new EPL establishes for the first time an environmental public interest litigation system, enabling environmental NGOs to file environmental public interest lawsuits directly against polluting firms, greatly reducing their litigation costs. Concerning the role of public supervision of the environment, Stafford [37] details how environmental NGOs have changed the perceptions of governments and international agencies on environmental issues, used public opinion and expertise to promote environmental protection legislation, and actively assisted in enforcement strategies. Fazin and Bond [38] argue that expanding civil rights and enhancing public supervision can help improve the efficiency of environmental policymaking, reduce corporate pollution, and improve local environmental quality.

Due to the new EPL implementation, the cost of public supervision, including environmental NGOs, has been reduced, which helps to enhance the monitoring of high polluting firms. Environmental supervision of high polluting firms will be increased because pollution information must be disclosed to the public. Furthermore, firms will be more likely to adopt proactive environmental strategies. Environmental legislation is positive interaction with public supervision. Based on the above discussion, we propose the following hypothesis:

***H2b*: *The association between high polluting firms and the capitalization of corporate environmental expenditures is more significant for firms in areas with higher public supervision than for firms with lower public supervision*, *subsequent to the new Environmental Protection Law (EPL) implementation*.**

We investigate the impact of financing constraints and managerial myopia on the capitalization of environmental expenditures by high polluting firms, as the change in capitalization may be more pronounced for those firms with more significant internal resource constraints after the new EPL implementation.

In terms of firms’ internal resources, the capitalization of firms’ environmental protection inputs is affected by their financing constraints. Firms’ investment expenditures depend on their financing constraints [39,40], and when firms are financially constrained, their investment expenditures are reduced. Compared to environmental capital expenditures, firms’ environmental expense expenditures require less capital and have a lower impact ontheir current cash flows. A firm with fewer financing constraints has sufficient cash to invest in environmental protection. The firm has a higher capitalization of corporate environmental expenditures.

Conversely, when a firm faces more financing constraints and insufficient cash flow,it prefers to choose environmental expense expenditures over capital expenditures, and the firm has a lower capitalization of corporate environmental expenditures. After the implementation of the new EPL, high polluting firms with fewer financing constraints capitalize their environmental expenditures more significantly, as they have sufficient resources to prevent future environmental risks. Therefore, we propose the following hypothesis:

***H3a*: *The association between high polluting firms and the capitalization of corporate environmental expenditures is more significant for firms with lower financing constraints than firms with higher financing constraints*, *subsequent to the new Environmental Protection Law (EPL) implementation*.**

From the perspective of corporate managers, the capitalization of corporate environmental expenditures is affected by the level of managerial myopia. Research shows that managerial myopia influences corporate behavior [41] and leads to the neglect of long-term strategy by focusing on the firm’s current strategy [42]. The effect of managers on corporate environmental performance is significant. According to Herremans and Nazari [17], the stringency and characteristics of environmental disclosures depend on management motivations and attitudes.

Those firmswith higher managerial myopia will choose negative environmental strategies since they emphasize short-term performance and ignore long-term environmental risks. Compared to capitalizing on corporate environmental expenditures, expensing them has a more negligible impact on the firm’s current profit. The manager with more myopia is more likely to choose to expense corporate environmental expenditures. In contrast, the manager is lower myopia and focuses on sustainable development, and is more inclined to choose environmental capital expenditures. After implementing the new EPL, high polluting firms with lower managerial myopia tend to capitalize corporate environmental expenditures. Therefore, we propose the following hypothesis:

***H3b*: *The association between high polluting firms and the capitalization of corporate environmental expenditures is more significant for firms in areas with lower managerial myopia than for firms with higher managerial myopia*, *subsequent to the new Environmental Protection Law (EPL) implementation*.**

## 3. Data and methodology

### 3.1 Data and sample

To test our hypotheses, we manually collected data on corporate environmental protection expenditures from financial reports and data from CSMAR and CNRDS databases for listed companies in China’s heavy pollution industry from 2011 to 2020. Our sample period starts in 2011 because (i) the information disclosed in previous annual reports is not sufficient to collect our main variables; and (ii) we need an appropriate period (e.g., 4 years) for DID estimation until 2015 (the first year of the new EPL implementation). We exclude: (i) listed companies that do not disclose their corporate environmental expenditures in their annual reports, such as companies that do not disclose both capital and expense corporate environmental expenditures; and (ii) companies that do not have financial variables. Then, in relation to local environmental enforcement (Punish), public supervision (EnvNGO), financing constraints (KZ), managerial myopia (Myopia), and the control variables (Size, Lev, Growth, Roa, Age, Ocf, Subsidy, State, First, INIHold, Board, Indr, and Dual, HHI, and Market) generated by the other merged missing values were excluded when merging the missing values. The data sample is 6,295 entries, the primary sample for the main regression, the main robustness test, and the subsample test. The key continuous variables are winsorized at the 1% level to eliminate the influence of extreme values.

### 3.2 Measures of capitalization of corporate environmental expenditure

The capitalization or expense of a firm’s expenditures is an accounting policy choice that can impact the firm’s investment opportunities and investment efficiency [43]. As mentioned earlier, the capitalization of corporate environmental expenditures is indicative of the corporate environmental strategy. Referring to prior studies [11–13], environmental capital expenditures can directly reflect environmental actions. Unlike the existing literatures that focus only on environmental capital expenditures, we reflect the corresponding environmental strategies by distinguishing between the environmental capital expenditures and the environmental expense expenditures.

We manually collect firm-level environmental capital expenditures under "construction in progress" in firms’ annual reports. We identify investment projects related to environmental protection from the detailed items under this account (e.g., desulfurization projects, denitrification projects, wastewater treatment, waste gas, dust, energy saving or greening projects, etc.). We then aggregate all relevant investments into the company’s environmental capital expenditures.

Similarly, we manually collect company-level environmental expenditures under "Administrative expenses" and "Taxes and surcharges" in the company’s annual report, and we identify environmental protection-related expense items (e.g., sewage charges, environmental protection taxes, etc.) from the detailed items under these accounts. We then aggregated all related expenses into the company’s environmental expense expenditures.

Finally, combining environmental capital expenditures and expense expenditures, we take the ratio of environmental capital expenditures to the sum of environmental capital expenditures and expense expenditures to measure the capitalization of corporate environmental expenditures. The larger value for capitalization of corporate environmental expenditures (EnvCapital), the more inclined a firm is to choose environmental capital expenditures and to have a poactive environmental strategy.

### 3.3 Difference-in-difference tests

Following Zhang et al. [13], we use the difference-in-difference method to study the impact of the new EPL implementation on the capitalization of corporate environmental expenditures. Implementing the new EPL has increased the penalties for environmental violations and the transparency of environmental information for polluting firms. Environmental legislation provides a natural quasi-experiment to examine changes in the capitalization of corporate environmental expenditures for two types of firms: treatment firms and control firms. We treat high polluting firms that are more affected by environmental legislation as treatment firms, whereas low polluting firms that are less affected by environmental legislation as control firms. The key to identifying a high polluting firm is whether the firm is included in the MEE’s National Special Monitoring Firms (NSMF) program. Firms with high emissions will be included in the program as high polluters for the year. The DID method compares the average change in capitalization of environmental expenditures over time for treatment firms with the average change over time for control firms. If environmental legislation facilitates public supervision and thus induces firms to adopt proactive environmental strategies, one would expect that treatment firms would have a higher capitalization of corporate environmental expenditures following environmental legislation compared to control firms.

### 3.4 Baseline model

We use a difference-in-differences (DID) model to test our main hypothesis about the relationship between environmental legislation and corporate capitalization of environmental expenditures. This model will also be applied to further analysis of hypotheses 2a as well as 2b and hypotheses 3a and 3b.

For Eq (1), the dependent variable is capitalization of corporate environmental expenditures measured by EnvCapital. The variable of interest is Treat×Post, and the coefficient β2 reflects the change in capitalization of corporate environmental expenditures for treatment firms compared to control firms after the new EPL implementation.


$$EnvCapital=\beta_{0}+\beta_{1}Treat_{i,t}\times Post_{i,t}+\gamma Control_{i,t}+\Sigma Year+\Sigma Firm+\Sigma Province+\varepsilon_{i,t}$$ (1)

We include several firm characteristics as controls that previous studies have found to be associated with corporate environmental expenditures [12,13]. These characteristics include firm size (Size), financial leverage (Lev), growth (Growth), profitability (Roa), listing years (Age), operating cash flow (Ocf), environmental subsidies (Subsidy), and nature of property rights (State). Also, corporate governance in terms of majority shareholder ownership (First), institutional investor ownership (INIHold), board size (Board), the proportion of sole directors (Indr), and dual positions (Dual). As well as the Herfindahl–Hirschman Index (HHI) controls industry competition based on operating income, and the Marketability Index for regions (Market). Year, Firm and Province are proxies for the dummy variables that account for year, firm, and region fixed effects. Control_i,t_ are control variables, and ɛ_i,t_ is the error term. In addition, firm-level standard error clustering was used to mitigate concerns about heteroscedasticity. A detailed description of the variables is provided in Table 1.

**Table 1:** Variable definition.

***Dependent variables***	
*EnvCapital*	The ratio of environmental capital expenditures to the sum of corporate environmental capital expenditures and expense expenditures
***Independent variable***	
*Treat*	a dummy variable that equals 1 the company is included in the National Special Monitoring Firm (NSMF) program
*Post*	a dummy variable that equals 1 for periods after 2015
***Other variables***	
*Size*	The natural logarithm of total assets at the end of the year
*Lev*	The ratio of total liabilities to total assets at the end of the year
*Growth*	Annual revenue growth rate from year
*Roa*	The ratio of annual net profit to total assets at the end of the year
*Age*	The number of firm listing age
*Ocf*	Ratio of cash from operation to total assets
*Subsidy*	log of 1 plus the number of environmental government subsidies plus 1
*State*	A dummy variable that equals 1 if the firm is a state-owned enterprise, and otherwise 0.
*First*	The shareholding ratio of the largest shareholder at the end of the year
*INIHold*	The proportion of the firm’s shares held by institutional investors
*Board*	Natural logarithm of number of directors sitting on the board
*Indr*	The percentage of independent directors on the board
*Dual*	A dummy variable that equals 1 if the chairman and the CEO are the same person and 0 otherwise
*HHI*	Calculated by squaring the operating income share of each firm in the industry and adding all firms up
*Market*	FanGang Index, refer to ‘China Market Index’
*Punish*	Number of environmental administrative penalties divided by industrial output (millions of Yuan)
*EnvNGO*	The number of Environmental NGOs divided by population (millions)
*FC*	KZ index, following Kaplan and Zingales [40]
*Myopia*	A dummy variable that equals 1 if firms’ R&D expenditures shrank this year compared to the previous year, following Chen et al. [41]

## 4. Empirical results

### 4.1 Descriptive statistics and correlations

Panel A of Table 2 provides summary statistics for the main variables used in the analysis. The mean and median of EnvCapital are 0.478 and 0.488, respectively, which indicates that corporate environmental capital expenditures account for roughly 47.8% of the sum of corporate environmental capital expenditures and expense expenditures. The standard deviation of EnvCapital is 0.455, which indicates there are significant firm differences in the capitalization of corporate environmental expenditures. The mean value of Treat is 0.499, and the median value is 0, indicating that almost half of the firms in the sample are under national special monitoring. We describe several notable statistics in relation to the independent variables to demonstrate the composition of the total sample. The mean value of Size is 22.336, and the average firm’s leverage is 0.423. The sample firms have experienced revenue growth at a mean value of 0.130, and the average firm’s net profit is accounting for 3.8% of total assets. the average number of Ocf is 0.060 and 39.5% of the sample firms are state-owned enterprises. The mean value of subsidies received by firms is 7.483, with a range from 0 to 18.561.

**Table 2:** Descriptive statistics.

Panel A															
Variable		N		Mean		SD			Min			p50		Max	
*EnvCapital*		6.295		0.478		0.455			0.000			0.488		1.000	
*Treat*		6.295		0.499		0.500			0.000			0.000		1.000	
*Size*		6.295		22.336		1.257			19.879			22.168		26.209	
*Lev*		6.295		0.423		0.204			0.035			0.414		0.979	
*Roa*		6.295		0.038		0.063			-0.445			0.036		0.245	
*Growth*		6.295		0.130		0.308			-0.579			0.082		2.354	
*Age*		6.295		11.081		6.985			0.000			10.000		27.000	
*Ocf*		6.295		0.060		0.066			-0.173			0.058		0.283	
*Subsidy*		6.295		7.483		7.049			0.000			10.690		18.561	
*State*		6.295		0.395		0.489			0.000			0.000		1.000	
*First*		6.295		0.356		0.145			0.078			0.338		0.825	
*INIHold*		6.295		0.058		0.064			0.000			0.037		0.410	
*Board*		6.295		2.152		0.196			1.609			2.197		2.708	
*Indr*		6.295		0.372		0.052			0.286			0.333		0.571	
*Dual*		6.295		0.244		0.430			0.000			0.000		1.000	
*HHI*		6.295		0.077		0.067			0.014			0.065		0.611	
*Market*		6.295		7.714		1.866			1.020			7.880		10.000	
Panel B															
Variables	Treatment group							Control group							
	Before adoption		Post- adoption		Difference			Before adoption		Post- adoption	Difference				Difference in difference
*EnvCapital*	0.479		0.646		-0.167***			0.353		0.363	-0.010				0.157***

Panel B of Table 2 shows the univariate tests on the change in corporate environmental strategies. In the post-adoption period, treatment firms increase the capitalization of corporate environmental expenditures (from 0.479 to 0.646, the difference is significant at the 1% level), but there is no significant change for control firms. This result indicates that the policy has a more significant effect on the treatment group than the control group. The results provide preliminary evidence that the implementation of new EPL is correlated with higher capitalization of corporate environmental expenditures. This preliminary observation also supports our analysis using DID approach.

Table 3 presents the correlation matrix of our key variables, with Pearson’s (Spearman’s rank) correlation coefficients in the lower (upper) triangle. Consistent with the above univariate tests, the variable EnvCapital is positively correlated with our proxy of Treat. The Pearson and Spearman correlation coefficients are 0.261 and 0.237 (significant at the 1% level), respectively. It indicates that high polluting firms capitalize corporate environmental expenditures. Control variables Size, Lev, Age, Ocf, Subsidy, and State are positively related with variable EnvCapital. It provides good rational and tentative support for the consolidation between the variables. The relatively low correlation coefficients between the variables show that the colinearity problem is not of concern.

**Table 3:** Correlation matrix of selected variables (Pearson\Spearman).

	*EnvCapital*	*Treat*	*Size*	*Lev*	*Roa*	*Growth*	*Age*	*Ocf*	*Subsidy*	*State*	*First*	*INIHold*	*Board*	*Indr*	*Dual*	*HHI*	*Market*
*EnvCapital*	1	0.237***	0.182***	0.112***	-0.028**	-0.004	0.123***	0.028**	0.128***	0.175***	0.057***	0.023*	0.058***	-0.005	-0.079***	0.036***	-0.061***
*Treat*	0.261***	1	0.427***	0.299***	-0.112***	-0.087***	0.337***	0.107***	0.258***	0.330***	0.075***	0.050***	0.178***	-0.042***	-0.148***	0.030**	-0.165***
*Size*	0.204***	0.427***	1	0.489***	-0.069***	0.005	0.426***	0.113***	0.295***	0.380***	0.216***	0.281***	0.268***	0.002	-0.195***	0.157***	-0.154***
*Lev*	0.117***	0.293***	0.466***	1	-0.476***	-0.041***	0.328***	-0.128***	0.261***	0.324***	0.053***	0.007	0.181***	-0.010	-0.116***	0.141***	-0.196***
*Roa*	-0.005	-0.061***	-0.002	-0.428***	1	0.354***	-0.211***	0.446***	-0.138***	-0.231***	0.065***	0.260***	-0.053***	-0.007	0.051***	-0.118***	0.164***
*Growth*	0.003	-0.071***	0.011	-0.018	0.288***	1	-0.176***	0.075***	-0.052***	-0.108***	-0.010	0.181***	-0.038***	0.022*	0.036***	-0.065***	0.032**
*Age*	0.137***	0.331***	0.392***	0.321***	-0.137***	-0.122***	1	0.052***	0.186***	0.483***	-0.028**	0.047***	0.148***	0.001	-0.224***	0.107***	-0.236***
*Ocf*	0.035***	0.108***	0.114***	-0.133***	0.422***	0.050***	0.058***	1	0.040***	-0.003	0.097***	0.140***	0.025**	-0.002	-0.037***	-0.010	0.068***
*Subsidy*	0.118***	0.216***	0.224***	0.216***	-0.076***	-0.033***	0.144***	0.025**	1	0.248***	0.071***	0.060***	0.153***	-0.054***	-0.140***	0.098***	-0.200***
*State*	0.176***	0.330***	0.398***	0.327***	-0.148***	-0.091***	0.479***	-0.004	0.213***	1	0.221***	0.009	0.268***	-0.028**	-0.280***	0.132***	-0.309***
*First*	0.061***	0.075***	0.255***	0.053***	0.090***	-0.004	-0.023*	0.102***	0.052***	0.226***	1	-0.031**	0.021	0.016	-0.060***	0.166***	-0.070***
*INIHold*	-0.012	0.011	0.178***	-0.013	0.229***	0.119***	0.030**	0.127***	0.015	-0.021	-0.081***	1	0.066***	0.021*	-0.013	-0.008	-0.000
*Board*	0.071***	0.181***	0.296***	0.196***	-0.015	-0.042***	0.150***	0.025**	0.125***	0.285***	0.042***	0.044***	1	-0.531***	-0.156***	0.080***	-0.150***
*Indr*	-0.003	-0.038***	0.004	-0.018	0.002	0.019	0.009	0.011	-0.057***	-0.024*	0.028**	0.011	-0.495***	1	0.079***	0.005	-0.000
*Dual*	-0.085***	-0.148***	-0.184***	-0.114***	0.022*	0.024*	-0.218***	-0.032**	-0.126***	-0.280***	-0.074***	0.010	-0.152***	0.073***	1	-0.088***	0.159***
*HHI*	0.005	0.004	0.131***	0.089***	-0.066***	-0.029**	0.092***	-0.016	0.023*	0.122***	0.142***	-0.044***	0.066***	-0.008	-0.064***	1	-0.110***
*Market*	-0.065***	-0.167***	-0.142***	-0.187***	0.100***	0.013	-0.222***	0.053***	-0.174***	-0.292***	-0.069***	0.010	-0.140***	-0.002	0.149***	-0.131***	1

This table reports a correlation matrix of the key variables. Pearson’s correlation coefficients are shown in the lower triangle, including the diagonal, while Spearman’s rank correlations appear above the diagonal.

***, **, and * indicate that the estimated coefficients are statistically significant at the 1%, 5%, and 10% level, respectively, based on two-tailed tests.

### 4.2 Regression results of baseline model

We predict that high polluting firms will increase the capitalization of corporate environmental protection expenditures, subsequent to the new Environmental Protection Law. Table 4 reports our main results. Column (1) is the regression result of Eq (1) without adding any control variables and fixed effects, and columns (2) and (3) gradually add year, firm, province fixed effects and control variables on this basis. The Adjusted R Square gradually increases, from 0.074 to 0.487. It indicates the enhancement of the goodness of fit of the model and the robustness of the results. The coefficients of the Treat×Post are significantly positive at the 1% level. This suggests that high polluting firms have made more environmental capital expenditures after implementing the new EPL. The results support hypothesis 1. This also suggests firms face heterogeneous environmental regulations with different strategies for dealing with them, with firms facing stronger environmental regulation adopting higher-quality environmental investment strategies.

**Table 4:** The new environmental protection law and the capitalization of corporate environmental protection expenditures.

	(1)	(2)	(3)
Variable	*EnvCapital*	*EnvCapital*	*EnvCapital*
***Treat×Post***	0.259***	0.074***	0.074***
	(16.467)	(3.320)	(3.328)
*Size*			0.046*
			(1.931)
*Lev*			-0.063
			(-0.782)
*Roa*			0.232*
			(1.843)
*Growth*			0.010
			(0.525)
*Age*			0.072
			(0.479)
*Ocf*			-0.091
			(-0.884)
*Subsidy*			0.000
			(0.115)
*State*			0.069
			(1.102)
*First*			-0.027
			(-0.214)
*INIHold*			0.122
			(0.950)
*Board*			-0.014
			(-0.180)
*Indr*			0.128
			(0.507)
*Dual*			-0.022
			(-1.009)
*HHI*			0.092
			(0.369)
*Market*			-0.014
			(-0.500)
*Constant*	0.387***	0.331***	-1.035
	(32.123)	(5.003)	(-0.939)
Year	N	Y	Y
Firm	N	Y	Y
Province	N	Y	Y
Observations	6,295	6,295	6,295
Adjusted R^2^	0.074	0.485	0.487

Note: The t-statistics reported in parentheses are calculated based on robust standard errors clustered by the company.

***, **, and * indicate significance at the 1%, 5%, and 10% two-tailed levels, respectively. The same as in the following table.

### 4.3 Robustness checks

#### (1) Parallel trend test

Parallel trends in the treatment and control groups are necessary for the effectiveness of the DID method, which requires that the treatment group and the control group have a stable level of the firm’s environmental capital expenditures before implementing the new EPL. Following Bertrand and Mullainathan [44], Fang et al. [45], and Zheng et al. [46], an event study method is used to test whether the parallel trend is satisfied and also to reflect the dynamic effect of the implementation of the new EPL.

**Fig 1:**
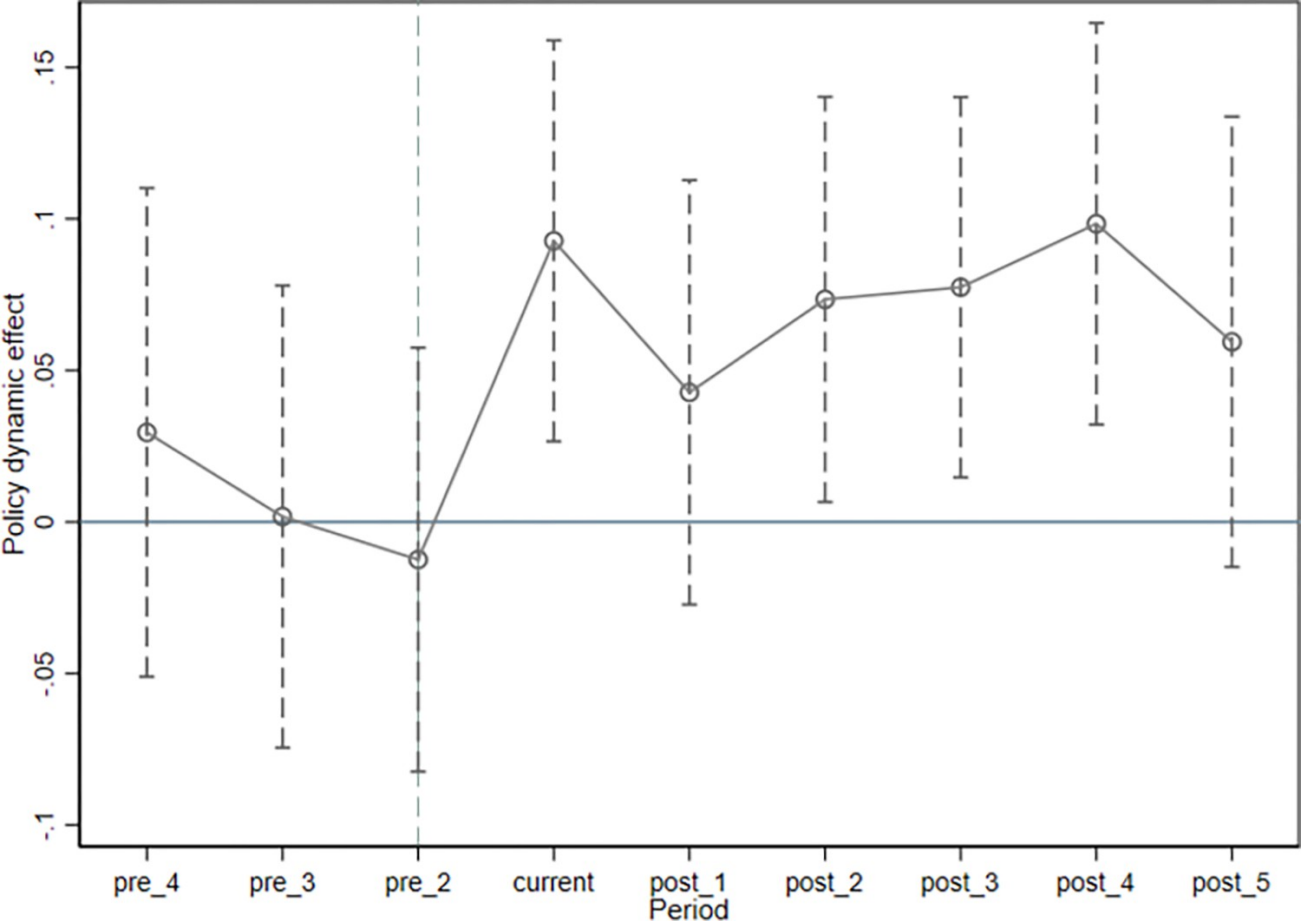
Parallel trend test.

As shown in Fig 1, the parallel trend hypothesis test results show that the significance levels of Before2, Before3, and Before4 regression coefficients of crossing item (Treat×Post) are all greater than 10%, which is not significantly different from zero. This shows that the Parallel Trend hypothesis is valid. That is, the control group and the treatment group are comparable before the new EPL.

#### (2) Alternative measures of firms’ environmental investment strategies

We examine whether our main findings are robust to using alternative measures of firms’ environmental investment strategies, EnvInvest. EnvInvest is a dummy variable that equals 1 if the firm has environmental capital expenditures in year t and 0 otherwise. The regression results are shown in Table 5. By sequentially adding year, firm, and province fixed effects and control variables, the Pseudo R Square gradually increases, from 0.070 to 0.254. It indicates the enhancement of the goodness of fit of the model and the robustness of the results. Consistent with the previous results, the coefficient of the crossing item (Treat×Post) is positively significant at the 1% level.

**Table 5:** Alternative measures of firms’ environmental investment strategies.

	(1)	(2)	(3)
Variable	*EnvInvest*	*EnvInvest*	*EnvInvest*
***Treat×Post***	0.840***	0.388***	0.394***
	(17.414)	(3.517)	(3.572)
*Size*			0.158
			(1.292)
*Lev*			-0.468
			(-1.227)
*Roa*			0.507
			(0.699)
*Growth*			0.060
			(0.534)
*Age*			0.128
			(0.267)
*Ocf*			-0.011
			(-0.018)
*Subsidy*			0.000
			(0.038)
*State*			0.417
			(1.342)
*First*			-0.035
			(-0.052)
*INIHold*			0.626
			(0.856)
*Board*			-0.389
			(-0.999)
*Indr*			0.296
			(0.254)
*Dual*			-0.037
			(-0.319)
*HHI*			-0.795
			(-0.496)
*Market*			-0.153
			(-1.117)
*Constant*	-0.213***	-0.558	-1.114
	(-6.353)	(-0.457)	(-0.289)
Year	N	Y	Y
Firm	N	Y	Y
Province	N	Y	Y
Observations	6,295	6,295	6,295
Pseudo R^2^	0.070	0.250	0.254

#### (3) Tobit regression method

Considering the characteristics of the data with left truncation (0 at truncation) for the dependent variable, the Tobit regression method is used for the robustness test. Regression results are shown in Table 6. By sequentially adding year, firm, and province fixed effects and control variables, the Pseudo R Square gradually increases, from 0.042 to 0.461. It indicates the enhancement of the goodness of fit of the model and the robustness of the results. The coefficients of Treat×Post keep significantly positive, which are consistent with the previous results.

**Table 6:** Tobit regression.

	(1)	(2)	(3)
Variable	*EnvCapital*	*EnvCapital*	*EnvCapital*
***Treat×Post***	0.448***	0.088***	0.089***
	(17.099)	(2.661)	(2.740)
*Size*			0.065*
			(1.819)
*Lev*			-0.101
			(-0.822)
*Roa*			0.332*
			(1.664)
*Growth*			0.028
			(0.944)
*Age*			0.064
			(0.343)
*Ocf*			-0.146
			(-0.900)
*Subsidy*			0.000
			(0.120)
*State*			0.136
			(1.491)
*First*			-0.154
			(-0.850)
*INIHold*			0.124
			(0.614)
*Board*			0.012
			(0.099)
*Indr*			0.319
			(0.878)
*Dual*			-0.039
			(-1.140)
*HHI*			0.031
			(0.080)
*Market*			-0.007
			(-0.213)
*Constant*	0.114***	0.849***	-1.944
	(4.245)	(5.055)	(-0.539)
Year	N	Y	Y
Firm	N	Y	Y
Province	N	Y	Y
Observations	6,295	6,295	6,295
Pseudo R^2^	0.042	0.458	0.461

#### (4) PSM

Considering the problem that the focus-monitored firms may naturally differ from others, thus resulting in the conclusions of this paper suffering from sample selection bias, we adopt the propensity score matching (PSM) method to address this potential issue. We based on a one-to-one, the nearest neighbor matching principle to construct the treatment and control groups for high polluting firms. The result presented in Table 7, by sequentially adding year, firm, and province fixed effects and control variables, the Adjusted R Square gradually increases, from 0.072 to 0.583. It indicates the enhancement of the goodness of fit of the model and the robustness of the results. The main findings of this paper remain valid after the propensity score matching screening of the sample.

**Table 7:** PSM result.

	(1)	(2)	(3)
Variable	*EnvCapital*	*EnvCapital*	*EnvCapital*
***Treat×Post***	0.257***	0.075***	0.074***
	(16.155)	(3.318)	(3.334)
*Size*			0.043*
			(1.773)
*Lev*			-0.053
			(-0.655)
*Roa*			0.237*
			(1.845)
*Growth*			0.012
			(0.585)
*Age*			0.071
			(0.471)
*Ocf*			-0.101
			(-0.964)
*Subsidy*			0.000
			(0.238)
*State*			0.069
			(1.092)
*First*			-0.042
			(-0.326)
*INIHold*			0.122
			(0.957)
*Board*			-0.016
			(-0.198)
*Indr*			0.109
			(0.425)
*Dual*			-0.021
			(-0.962)
*HHI*			0.102
			(0.404)
*Market*			-0.013
			(-0.467)
*Constant*	0.386***	0.329***	-0.953
	(32.061)	(4.924)	(-0.868)
Year	N	Y	Y
Firm	N	Y	Y
Province	N	Y	Y
Observations	6,208	6,208	6,208
Adjusted R^2^	0.072	0.581	0.583

### 4.4 Additional analysis: External legitimacy pressure and internal resource

Based on the varying levels of local environmental enforcement in the regions where the firms are located, we divide the total sample into two subsamples, firms with strong local environmental enforcement and firms with weak local environmental enforcement. We then run separate regressions for the two subsamples. The results for the main variables are reported in Columns (1) and (2) of Table 8, where the Treat×Post coefficient is significantly positive for firms with stricter local environmental enforcement. The Adjusted R Square are 0.542 and 0.506, respectively. It indicates the goodness of fit of the model and is consistent with the previous results. The findings suggest that the positive effect of environmental legislation on the capitalization of environmental expenditures of high polluting firms is more significant in regions with higher environmental enforcement. Firms choose more proactive environmental strategies to gain legitimacy.

**Table 8:** Additional analysis: External legitimacy pressure.

	(1)	(2)	(3)	(4)
Variable	*EnvCapital*	*EnvCapital*	*EnvCapital*	*EnvCapital*
	*strict local environmental enforcement*	*weak local environmental enforcement*	*high public supervision*	*low public supervision*
***Treat×Post***	0.143***	**0.041**	0.114***	**0.041**
	**(4.880)**	**(0.968)**	**(3.476)**	**(1.275)**
*β1 difference*	0.102***		0.073**	
*Constant*	-1.459	0.123	-0.929	-1.092
	(-0.973)	(0.124)	(-0.668)	(-1.264)
*Controls*	Y	Y	Y	Y
Year	Y	Y	Y	Y
Firm	Y	Y	Y	Y
Province	Y	Y	Y	Y
Observations	3,331	2,964	3,366	2,929
Adjusted R^2^	0.542	0.506	0.537	0.512

Based on the varying intensity of public scrutiny in the regions where the firms are located, we divide the total sample into two subsamples, firms in regions with high public supervision and firms in regions with low public scrutiny. We then run separate regressions for the two subsamples. Columns (3) and (4) of Table 8 report the results for the main variables, and the Treat×Post coefficient is significantly positive for the firms facing higher public supervision. The Adjusted R Square are 0.537 and 0.512, respectively. It indicates the goodness of fit of the model and is consistent with the previous results. According to the findings, high polluting firms in regions with strong public supervision are more likely to capitalize their environmental expenditures as a result of environmental legislation. Firms choose more proactive environmental strategies to comply with stakeholders’ requirements.

In terms of internal resources constraints, corporate environmental expenditures are affected by their operating cash flows. Based on the heterogeneity of financing constraints firms face, we divide the total sample into two subsamples: firms with low financing constraints and firms with high financing constraints. We then run separate regressions for the two subsamples. Columns (1) and (2) of Table 9 report the results for the main variables, and the Treat×Post coefficient is significantly positive for high polluting firms with low financing constraints. The Adjusted R Square are 0.472 and 0.541, respectively. It indicates the goodness of fit of the model and is consistent with the previous results. Consistent with existing studies, the findings suggest that firms’ investment in the environment is affected by their financing constraints [39], and firms with lower financing constraints have more resources available to capitalize corporate environmental expenditures.

**Table 9:** Additional analysis: Internal resource constraints.

	(1)	(2)	(3)	(4)
Variable	*EnvCapital*	*EnvCapital*	*EnvCapital*	*EnvCapital*
	*high financing constraints*	*low financing constraints*	*high managerial myopia*	*low managerial myopia*
***Treat×Post***	**0.045**	0.101***	**-0.001**	0.114***
	**(1.275)**	**(2.844)**	**(-0.008)**	**(4.606)**
*β1 difference*	-0.056*		-0.115***	
*Constant*	1.367	-2.988**	4.214	-1.222
	(1.644)	(-2.360)	(1.512)	(-1.067)
*Controls*	Y	Y	Y	Y
Year	Y	Y	Y	Y
Firm	Y	Y	Y	Y
Province	Y	Y	Y	Y
Observations	3,126	3,169	1,259	5,036
Adjusted R^2^	0.472	0.541	0.436	0.503

In light of inconsistent behavioral characteristics of corporate management, management influences corporate strategic decisions [41]. We divide the total sample into two subsamples, firms with low managerial myopia and firms with high managerial myopia. We then run separate regressions for the two subsamples. Columns (3) and (4) of Table 9 report the results for the main variables, and the Treat×Post coefficient is significantly positive for high polluting firms with low managerial myopia. The Adjusted R Square are 0.436 and 0.503, respectively. It indicates the goodness of fit of the model and is consistent with the previous results. According to the findings, managerial myopia negatively impacts corporate environmental strategies, and lower managerial myopia leads to higher capitalization of corporate environmental expenditures.

### 4.5 Plausible channels: Media attention and perceived environmental risk

The new EPL has increased the transparency of corporate environmental information disclosure, resulting in more media scrutiny of corporate environmental behavior. In addition, the new EPL has increased penalties, and firms’ perceptions of future environmental risks have risen. They will be more willing to choose environmental capitalization expenditures to prevent future risks. Therefore, this paper examines the possible impact mechanisms of the new EPL to increase the capitalization of corporate environmental expenditures in terms of both the environmental media attention that firms receive and their environmental risk perceptions.

Referring to Jia et al. [47], media attention (EnvMedia) is the number of environmental-related news media reports of the firm plus one, taken as a logarithm to measure the environmental-related media attention received by the firm. Compared to the old EPL, a critical category added to the new EPL is strengthening environmental information disclosure and public supervision. High polluting firms need to disclose their emission information and accept supervision from external organizations such as the media. Hence, this paper argues that high polluting firms may receive more attention from the media on their environmental information, thus prompting them to capitalize their environmental expenditures. As shown in columns (1) and (2) of Table 10, by sequentially adding year, firm, province fixed effects, and control variables, the Adjusted R Square gradually increases, from 0.400 to 0.401. It indicates the enhancement of the goodness of fit of the model and the robustness of the results. Our study focuses on the interaction between Treat×Post and media attention (EnvMedia). This coefficient is significantly positive at the 5% level, indicating that high polluting firms receive more media attention and experience higher external media supervision after the implemention of the new EPL.

**Table 10:** Plausible channels: Media attention and perceived environmental risk.

	(1)	(2)	(3)	(4)
Variable	*EnvMedia*	*EnvMedia*	*EnvRisk*	*EnvRisk*
***Treat×Post***	0.060**	0.060**	0.144***	0.143***
	**(2.202)**	**(2.167)**	**(2.881)**	**(2.879)**
*Constant*	0.313***	-0.470	1.250***	3.196
	(5.011)	(-0.375)	(4.720)	(1.340)
*Controls*	N	Y	N	Y
Year	N	Y	N	Y
Firm	N	Y	N	Y
Province	N	Y	N	Y
Observations	6,295	6,295	6,295	6,295
Adjusted R^2^	0.400	0.401	0.626	0.626

Referring to Chen et al. [48], the perceived environmental risk (EnvRisk) is the logarithm of one plus the number of environmental keywords in the "Management Analysis and Discussion" section of the annual report. The more environmental-related words disclosed in the annual report, the more attention is paid to environmental risk and the more environmental capital expenditures are made by firms. As shown in columns (3) and (4) of Table 10, the explanatory variable is perceived environmental risk (EnvRisk). By sequentially adding year, firm, province fixed effects, and control variables, the Adjusted R Square keep at 0.626. It indicates the level of the goodness of fit of the model and the robustness of the results. The coefficient of Treat×Post is significantly positive, indicating that high polluting firms attach more importance to managing their environmental risk after the new EPL implementation, and are more willing to adopt proactive environmental strategies to reduce this risk.

## 5 Conclusion

This study uses the implementation of the new EPL as a natural experiment to explore the impact of environmental legislation on polluting firms’ capitalization of environmental expenditures. In light of implementing the new EPL may increase the media attention and perceived environmental risks of high polluting firms, firms tend to choose a proactive environmental strategy, i.e., more environmental capital expenditures rather than environmental expense expenditures. As a result, we predict a positive relationship between environmental legislation and the capitalization of corporate environmental expenditures.The empirical findings of our study confirm our hypothesis that high polluting firms’ capitalization of environmental expenditures increases after the new EPL implementation, based on data collected manually from Chinese listed companies between 2011 and 2020. Further, we find that external legitimacy pressures, such as local environmental enforcement and public supervision, make the above relationship more significant. The above relationship is also more significant when firms face lower internal resource constraints,such as financing constraints and managerial myopia. The findings are consistent with the argument that firms strategically choose to spend on environmental protection in order to achieve legitimacy. We suggest that the positive impact of environmental legislation on the capitalization of environmental expenditures by high polluting firms is strong, and it helps them choose a proactive environmental strategy.

Our study has several theoretical and practical implications. First, this study adds to the environmental accounting literature an important, yet little-identified, measure of corporate environmental strategies, the capitalization of corporate environmental expenditures. According to the requirements of China’s MEE, listed companies in the polluting industriesmust fully disclose their environmental information, which provides an ideal context for manually identifying corporate environmental expenditures from financial reports. The manually collected data on environmental expenditures are more comprehensive than a single indicator, making our conclusions more convincing. Second, in exploring corporate environmental performance, we focus on the capitalization and expense of corporate environmental expenditures, providing empirical evidence for a new perspective on the impact of environmental regulation on corporate environmental performance. it contributes to the literature on the relationship between environmental regulation and corporate environmental performance. Due to the institutional background of the study and the availability of data, we only obtained results based on Chinese data, and future studies could explore international comparisons regarding the capitalization of corporate environmental expenditure. Finally, our findings will help polluting firms better to understand the impact of their choice of environmental strategies. Given the increasing scarcity of natural resources and environmental risks, it would be in the strategic interest of firms to actively manage environmental issues [49]. Firms need to increase their attention to environmental risks and adopt proactive environmental strategies to meet the demands of stakeholders such as the government, media, and the public to successfully gain legitimacy and help them achieve green and sustainable development.
